# Hydrolyzed Collagen for Skin Aging Meta‐Analyses Are Unreliable

**DOI:** 10.1111/jocd.71208

**Published:** 2026-09-28

**Authors:** David Robert Grimes, Anjali Mahto

**Affiliations:** ^1^ TCD Biostatistics Unit, Discipline of Public Health and Primary Care, School of Medicine Trinity College Dublin Dublin Ireland; ^2^ Self London London UK

## Abstract

**Background:**

Scientific evidence for oral collagen supplementation in skin aging remains controversial and limited, with various trials finding conflicting results on skin parameters such as reduction in wrinkle depth, increases in hydration and elasticity. These parameters are measured with a variety of methodologies, and recent meta‐analyses have attempted to synthesize existent evidence to ascertain potential benefits. The question of whether these measures are readily comparable, however, has led to concerns about the internal validity and interpretability of these efforts.

**Aims:**

To investigate major existing meta‐analyses on oral collagen supplementation and perform a technical re‐analysis of both methodology and reported results, as well as a full meta‐regression of constituent collagen studies accounting for dose effects to ascertain evidence quality.

**Methods:**

Data investigation and evidence appraisal of the methodology of existing meta‐analyses of collagen supplementation and implementation of meta‐regression accounting for cumulative dose effects.

**Results:**

In both identified meta‐analyses, we find inaccurate reporting of data from primary studies and inappropriate combinations of measures likely to give rise to spurious results. Meta‐regression of collagen studies accounting for dose variations in primary studies found no evidence of benefit from collagen supplementation.

**Conclusion:**

In sharp contrast to the reported results from prior meta‐analysis, this investigation does not find evidence of benefit for supplementation from existing collagen studies. Evidence synthesis for collagen studies needs a stronger methodological underpinning if results are to be clinically relevant.

## 1 Introduction

Collagen is the principal structural protein in the dermis, comprising about 80% of skin's dry weight and providing tensile strength and elasticity [1]. Age‐related decline in collagen production and fibroblast activity leads to progressive dermal thinning, loss of elasticity, dehydration, and wrinkle formation [2]. These biological changes have driven interest in interventions that might restore collagen and ameliorate skin aging. In recent years, oral hydrolyzed collagen supplements have surged in popularity as a purported antiaging nutraceutical. A growing number of nutraceutical companies market collagen peptides in powders and drinks, claiming benefits such as wrinkle reduction and improved skin hydration. This trend has been amplified by aggressive industry marketing and endorsements on social media, fueling consumer enthusiasm [3]. As a result, dermatologists are increasingly faced with patients who are using or inquiring about collagen supplements for skin health, and clinicians must critically assess the evidence behind these products.

Despite widespread commercial claims, the scientific evidence for oral collagen supplementation in skin aging remains controversial and limited. Small randomized controlled trials (RCTs) have been conducted, often sponsored by supplement manufacturers, to evaluate whether daily collagen peptide ingestion can improve skin appearance. Several of these trials report modest improvements in skin parameters such as reduction in wrinkle depth or increases in hydration and elasticity after 1–3 months of collagen use [4]. These findings have led to multiple systematic reviews and meta‐analyses concluding that hydrolyzed collagen supplementation produces statistically significant benefits for aging skin. Most recently, a 2021 meta‐analysis of 19 trials (1125 participants) reported that 90 days of oral collagen led to measurable reductions in wrinkles along with improved skin elasticity and hydration compared to placebo [4]. Similarly, a 2023 systematic review including 26 RCTs found significant pooled improvements in skin hydration and elasticity with collagen supplements [2]. At face value, such results suggest that collagen nutraceuticals may indeed counteract some aspects of skin aging.

However, there are substantial methodological concerns that temper these optimistic conclusions. The current body of evidence is fraught with heterogeneity and potential bias. Trials of collagen for skin vary widely in design and quality: study populations range from young adults to the elderly; intervention regimens differ in collagen source (e.g., fish, porcine, bovine, or chicken origin), daily dosage (from as low as ~0.25 g to as high as 10–12 g of collagen peptides), and treatment duration (from 2 to 12 weeks). Outcomes are assessed using diverse measures, from objective instruments for hydration and elasticity to subjective wrinkle grading, making it difficult to standardize results across studies. This variability has led to considerable between‐study heterogeneity in meta‐analytic analyses. Furthermore, many trials have been small (often < 100 participants) and of short duration, which limits their power to detect meaningful long‐term effects [3].

Perhaps most importantly, an overwhelming proportion of studies are industry‐funded or involve authors with commercial ties to collagen manufacturers, raising concerns about selective reporting and bias. Indeed, dermatologists and researchers have grown wary that the marketing‐driven popularity of collagen supplements may be outpacing the rigor of the underlying science. In light of these issues, a critical appraisal of the evidence is needed. This article examines the quality of existing clinical research on hydrolyzed collagen supplementation for skin aging, evaluates the reliability of reported benefits, and discusses whether current evidence is sufficient to support recommending these supplements in dermatologic practice.

## 2 Methodological Issues With Existing Meta‐Analyses

### 2.1 Concerning Levels of Heterogeneity

In the pooled analysis, de Miranda et al. [4] find ostensibly beneficial effects of collagen supplementation on skin hydration, skin elasticity, and skin roughness using standardized mean differences (SMDs) for all these measures respectively. However, there are extremely high indicators of heterogeneity, with $I^{2}$ values of 93%, 98%, and 93% respectively. Even in the author's subgroup analyses, this remains exceptionally high, ranging from a minimum of 76% to a maximum of 99%. In Pu et al. [2], heterogeneity is 70% for the hydration group and 84% for skin elasticity measurements. These high levels of heterogeneity are indicative of significant variation between studies, and suggest potential differences in the populations and interventions which require deeper inspection.

### 2.2 Inappropriate Use of SMDs

In both meta‐analyses, the authors pool incompatible measures in sometimes methodologically inappropriate ways. In Miranda et al., to quantify skin hydration effects, the authors initially perform a meta‐analysis of studies contrasting placebo and HC groups across nine studies. Of these, the authors identify five with the outcome measure micrometers, two with percentage change, and a further two with arbitrary units (AU) in patients. Similarly, the authors looked at studies of skin elasticity, using 14 studies which used measures including pressure (four studies), percentage change (six studies), and AUs (four studies) as outcome measures. Pu et al. includes many of the studies in Miranda et al. and performs a similar exercise in grouping differing outcomes. For all these studies, the authors calculated SMDs to combine and analyze results together. This implicitly assumes that SMD treats everything on a standardized scale and that differences in units can be “standardized away.”

This is wrong; however, as SMD only works as a basis for comparison when the underlying constructs being measured are the same, with variation in scale the main difference. This is not valid when the meaning or structure of the outcome differs; percentage change, for example, is bounded and behaves differently from unbounded AUs. Nor is percentage change in elasticity equivalent to raw change in Young's modulus. The use of SMD for comparative purposes requires similar variance across studies, but in devices with different metrics or AUs can have wildly different variability, rendering SMD unsuitable for cross‐comparison in these instances [5, 6]. This ultimately renders clinical interpretation impossible, as there is no clear way to determine what a pooled SMD across incompatible measurement systems even means and masks critical differences in outcome structure, variance, and clinical meaning.

### 2.3 Data and Analysis Errors

In Miranda et al., the authors of this work attempt to control for different metrics by performing subgroup analyses stratified by measurement, but these are plagued by data and analysis errors that confound conclusions, some of these errors we elucidate in the sections below. Similarly Pu et al. have several of the same errors that undermine the entire analysis. An overview of some of these is given in Table 1.

**TABLE 1:** Overview of data and analysis errors in de Miranda et al. and Pu et al.

Skin hydration data reporting errors in de Miranda et al.	
Misrepresentation of methodology	Subgroup analysis asserts five studies expressed skin hydration in micrometers. This is incorrect—none of the nine studies quoted do this. Instead, four use capacitance methods to measure relative skin hydration (measured in arbitrary units, AU) and one (Žmitek et al. [7]) uses conductance methods measured in microsiemens. In total, eight of the nine hydration studies used capacitance methods to measure skin hydration
Incorrect usage of percentage difference	Both studies the authors state as measuring percentage difference (Koizumi et al. [8], Schwartz et al. [9]) actually use conductance methods with the authors failing to convert reported percentage difference to AU
Failure to convert standard error to standard deviation	The meta‐analysis authors also treated standard error from one study as the standard deviation (Koizumi et al. [8]) which should have been converted prior to analysis to avoid false positive results
Missing data	A study including hydration analysis (Yoon et al. [10], nonsignificant finding) was used for skin elasticity, but missed in meta‐analysis on hydration
Skin elasticity data reporting errors in de Miranda et al.	
Inclusion of studies with no placebo control	Of the 14 included studies pertaining to skin elasticity, Proksch et al. [11] did not compare HC to placebo and should not have been included in analysis
Failure to distinguish standard error from standard deviation	The authors incorrectly treated standard error from one study as standard deviation (Genovase et al. [12])
Incorrect usage of percentage difference	Of the six studies the authors claim use percentage differences and four the authors claim used arbitrary units in subgroup analysis, all 10 actually used cutometer methods (8× R2, 1× R5, 1× R7). In total, 10 of the 13 eligible studies used suction‐based cutometer measurements and three pressure measurements
Skin hydration data reporting errors in Pu et al.	
Missing studies	Of the 18 studies the authors asserted to have included the hydration analysis, one of these cited in the meta‐analysis table was not included in the paper references (Lin et al. [13]) and was explicitly referred to as excluded in the paper's text
Inappropriate inclusion	One paper's hydration data was based on a survey question without any objective analysis (Evans et al. [14]) and should not have been included
Errors in effect size reporting	Confusion of percentage differences as effect sizes, most obviously with Yoon et al. [10], reported by Pu et al. report as highly significant in their meta‐analysis despite the original authors explicitly stating the result was not significant. Percentage conversion not considered for other studies (Jung et al. [15] and Miyanaga et al. [16])
Failure to convert standard error to standard deviation	The authors also mistakenly cited two papers reporting standard error the mean as standard deviation (di Cerbo et al. [17] and Koizuma et al. [8]), producing false positive results
Skin elasticity data reporting errors in Pu et al.	
Incorrect usage of percentage difference	Failure to convert percentage difference to measurement differences in some included papers (Koizumi et al. [8] and Yoon et al. [10])
Failure to distinguish standard error from standard deviation	Genovase et al. [12] was included without converting SEM to SD, skewing analysis
Incorrect numbers of subjects	For one study (Kim et al. [18]), the total initially enrolled subjects is reported in the table rather than the total analyzed subjects. In total, 14 of the 18 eligible studies used suction‐based cutometer measurements, three used pressure measurements (same as de Miranda et al.) and one used unclear modality (di Cerbo et al. [17])

### 2.4 Analysis Issues and Lack of Dose Correction

Aside from the fact that correcting the data entry issues markedly changes conclusions of the meta‐analyses, a more relevant issue was not considered appropriately in either analysis—that of HC dose effects. In de Miranda et al., daily collagen dose varied from 0.3 to 10 g/day, an over 33× fold difference. In Pu et al., it values from 0.248 g a day to 10 g, an over 40× variation. A standard meta‐analysis that ignores doses effectively treats all doses as equivalent, implicitly assuming that treatment effect is binary. This is highly unlikely to be true and confounds interpretation and introduces study heterogeneity. For this reason, the analysis presented in both de Miranda et al. and Pu et al. is likely misleading.

### 2.5 Potential Risk of Bias Issues

In de Miranda et al., the authors did not indicate whether studies were industry supported or conducted in their risk of bias ranking. In that work, of the nine hydration reporting studies, seven were industry sponsored. Neither of the two nonindustry sponsored studies found significant effects of HC on skin hydration, whereas four of the seven industry sponsored studies did. Of the 13 eligible studies reporting skin elasticity in that meta‐analysis, 10 were industry sponsored or conducted. None of the three nonindustry sponsored studies found significant effects of HC on skin elasticity, whereas four of the 10 industry sponsored studies did.

Similar issues were seen in Pu et al., where of the 16 hydration studies with objective evidence, 13 were industry‐conducted or sponsored. None of the three nonindustry funded studies found any significant effect, whereas six of the 13 industry studies did. For elasticity, 12 of the 17 studies were industry conducted or funded. Of these, five of six studies yielding a positive effect of HC were industry funded, whereas only two of the four studies finding a negative impact of HC were industry funded. In the remaining seven nonsignificant studies, five of which were industry funded.

## 3 Meta‐Regression With Corrected Data

For the reasons outlined in the prior section, a more rigorous approach to ascertain potential impact of collagen supplementation would be to perform a meta‐regression with either daily dose or cumulative dose over the study period as a continuous predictor.

To investigate whether the conclusion of these meta‐analyses would hold if considering dose, we took corrected data (with corrections from the issues listed in Table 1) from both meta‐analysis and the dose data reported in the original papers, performing a simple meta‐regression analysis for both daily dose and cumulative dose (the product of daily dose over the entire duration of the study). The number of weeks on treatment was also considered as a covariate.

For both hydration and skin elasticity in both meta‐analyses, we also performed the meta‐regression on all included studies from both meta‐analyses with SMD as a measure, once units were corrected. We further performed subgroup meta‐regression on studies grouped by comparable methodologies, using mean differences as a basis for comparison.

## 4 Results

As shown in Table 2, no analysis showed any significant differences between placebo and HC groups with dose from either meta‐analysis. Full data and relevant code are given in the Supporting Information. Additionally, we synthesized all unique results from both meta‐analyses to provide an updated meta‐regression of dose effects (16 unique studies on hydration, 18 on elasticity). These results, as shown in Figure 1 do not support the interpretation that HC supplementation is beneficial for either skin hydration or elasticity.

**TABLE 2:** Meta‐regression of HC/placebo differences with daily dose for both meta‐analyses.

Analyses	Meta‐regressions	Included studies	Dose coefficient	*p‐value*	$I^{2}$
de Miranda et al. [4]	Hydration studies				
	All eligible studies	9	0.03 (−0.10, 0.15)	0.66	74.5%
	Capacitance methods	8	0.03 (−0.10, 0.15)	0.69	77.3%
	Elasticity studies				
	All eligible studies	13	−0.10 (−0.67, 0.46)	0.72	99.0%
	Pressure methods	3	−4.89 (−21.53, 11.79)	0.57	99.5%
	Suction methods^a^	10	−0.09 (−0.30, 0.12)	0.41	92.6%
Pu et al. [2]	Hydration studies				
	All eligible studies	16	0.07 (−0.01, 0.14)	0.08	58.8%
	Capacitance methods	14	0.07 (−0.01, 0.15)	0.08	63.4%
	Elasticity studies				
	All eligible studies	18	0.02 (−0.35, 0.39)	0.92	98.6%
	Pressure methods	3	−4.89 (−21.53,11.79)	0.57	99.5%
	Suction methods^a^	14	0.04 (−0.14,0.22)	0.70	94.1%

^a^ Suction methods included R2, R5, and R7 measurements.

**FIGURE 1:**
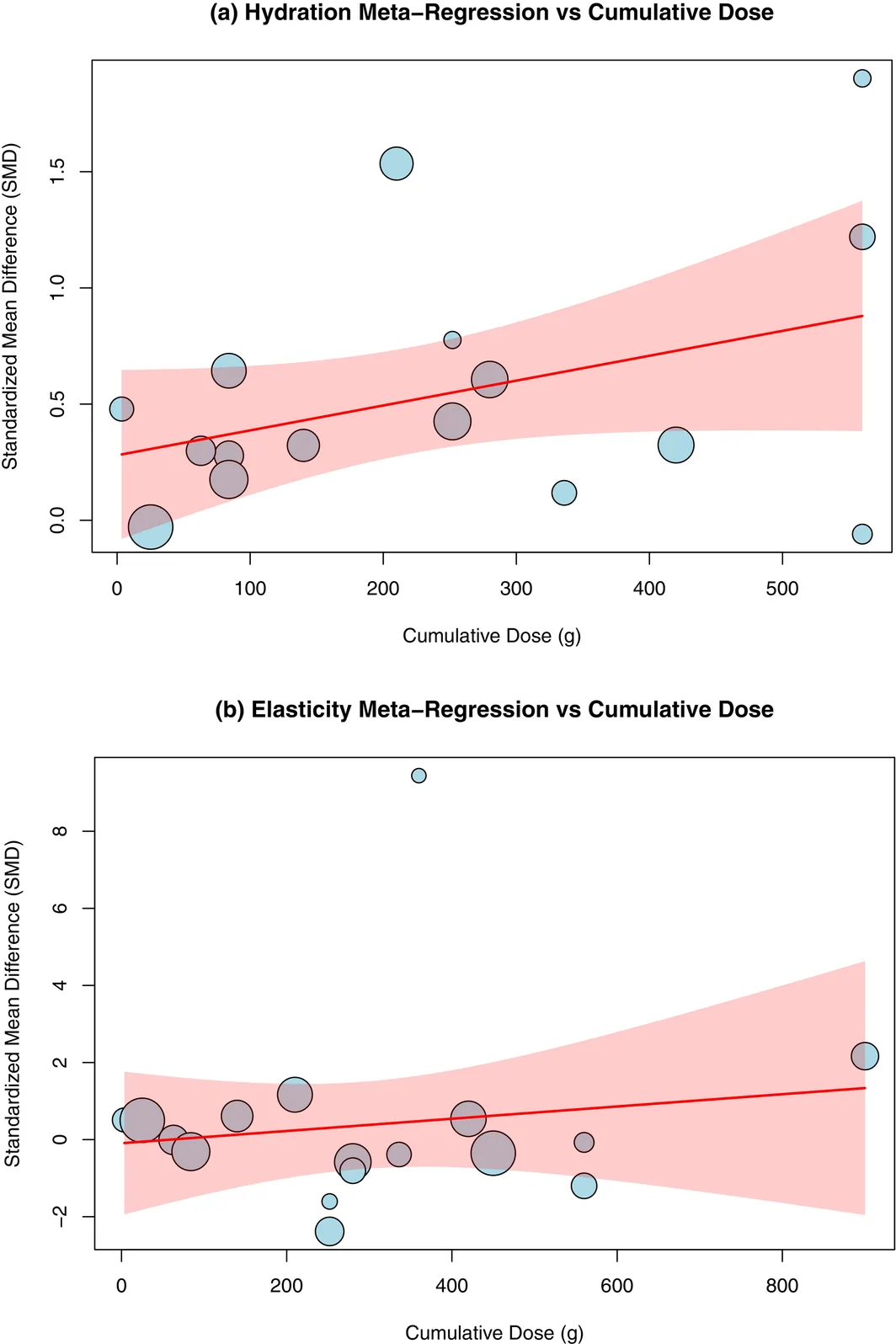
Meta‐regressions of all eligible studies in both meta‐analyses with dose as a covariate for (a) hydration studies (*p* = 0.08, $I^{2}=58.8\%$) and (b) elasticity studies (*p* = 0.92, $I^{2}=98.6\%$). Shaded region lines depict 95% confidence intervals, with circle size inversely proportional to study reported variability, where smaller circles indicate less variance. Vertical axis is standardized mean difference. Note that cumulative dose is shown here for illustration, but no significant relationships were found using daily dose or time on treatment in weeks as predictors.

## 5 Discussion

Despite the growing popularity of hydrolyzed collagen supplements as an intervention for skin aging, the current clinical evidence base remains methodologically fragile, inconsistent, and prone to bias. While several meta‐analyses have reported statistically significant improvements in hydration, elasticity, and skin roughness following oral collagen supplementation, the critical re‐evaluation outlined in this work reveals that many of these findings rest on flawed statistical assumptions, inadequate trial designs, and uncorrected sources of bias. A further limitation was that included studies in published meta‐analyses also include trials where collagen was not used in isolation, but as part of a multicomponent formulation with other bioactive ingredients. This limits not only the generalizability of results from these meta‐analyses, but applies to the synthesis in this work. It further underscores the reality that collagen meta‐analyses have underlying data problems in addition to the issues we discuss in this work. The inclusion of other potentially active agents alongside collagen may themselves affect aspects like skin hydration, elasticity, or appearance either positively or negatively. Previous meta‐analyses and investigations have made little effort to account for this methodological limitation, rendering it even more difficult to attribute observed effects specifically to hydrolyzed collagen. This is a methodological limitation future analyses should strive to avoid.

One of the most striking issues is the extreme heterogeneity across included studies. Previously published pooled analyses report *I* ^2^ values exceeding 90% for hydration, elasticity, and roughness outcomes [2]. In a closer re‐examination, this heterogeneity remained high even within subgroup analyses, raising concerns regarding the validity of the aggregated effect estimates. Much of this inconsistency arises from inappropriate pooling of fundamentally incompatible outcome measures using SMDs. In one widely cited meta‐analysis, skin hydration outcomes were combined from studies reporting in AUs, percentage change, and conductance (microsiemens), with no consideration given to the bounded nature or differential variance structures of these measures. Similarly, elasticity outcomes were pooled despite being measured via differing constructs: Young's modulus in megapascals (MPa), cutometry‐derived indices, and percentage changes in recoil time or deformation resistance. These metrics do not share the same underlying physiological interpretation and therefore cannot be considered interchangeable. This is not merely a difference in units of hydration or elasticity; it is ultimately an attempt to pool measures so physiologically distinct that the pooling of them represents a serious conceptual limitation. SMDs assume comparability of construct and distribution; this is invalid when the nature, clinical meaning, and scale of measurement differ so profoundly [19].

Subgroup analysis by measurement method demonstrates this clearly. In hydration studies, a reclassification of outcome metrics showed that eight of nine trials used capacitance‐derived AUs, and only one used conductance. Contrary to the original subgrouping, which incorrectly stated that five studies measured hydration in micrometers, none used this metric. A similar issue was identified in the elasticity domain. Of the 14 studies included in a prior meta‐analysis, one did not compare collagen to placebo and should have been excluded entirely, while another was mis‐analyzed due to incorrect variance calculations (using standard error instead of standard deviation). Additionally, the authors' categorization of elasticity measures into pressure‐based versus AU subgroups failed to acknowledge that 10 of the 13 valid studies used suction‐based cutometer measurements, specifically R2 (gross elasticity), R5 (net elasticity), or R7 (biological elasticity). These parameters are physiologically distinct: R2 represents total elastic recovery; R5 reflects the net elastic portion independent of viscous response; and R7 approximates the skin's ability to return to its original state after deformation. Pooling these without stratification obscures potential signal variance and introduces interpretative ambiguity. Notably, none of the included studies found consistent improvements across all three parameters; instead, findings were sporadic and method‐specific. The issue of dose variability also undermines pooled effect estimates. Across hydration and elasticity studies, collagen doses ranged from 0.3 to 10 g/day, i.e., an over 30‐fold difference.

The meta‐regression analyses in this work differed from previous undertakings by using dose as a continuous predictor (both daily and cumulative dose) which previous meta‐analysis had not considered despite its confounding influence. Meta‐regression showed no significant relationship between dosage and clinical outcome, either in mean difference models or SMD‐based comparisons. Most strikingly, there is an absence of a dose–response relationship that questions the plausibility of a genuine pharmacodynamic effect. It suggests that observed differences may be attributable to bias, noise, or behavioral confounders rather than any true biological gradient. If collagen's effects were mechanistically driven, one would anticipate a correlation between dose intensity and magnitude of change, yet this was not observed. This is a distinct strength of meta‐regression over meta‐analysis, and as the treatment time varied in the constituent trials, we regressed with respect to both dose per treatment and cumulatively over the course of treatment, finding no dose effects under either model. This work is the first to our knowledge to control for dose in regression, and that it fails to find any dose dependent effect suggests that ostensible benefits reported in previous meta‐analysis were likely spurious findings based on using a methodology that did not account for dose.

Bias related to industry sponsorship further clouds interpretation. Among hydration studies, seven of nine were funded by commercial manufacturers. Of these, four reported statistically significant results, whereas neither of the two independent studies did. Similar patterns were noted in elasticity trials, where 10 of 13 studies were industry funded; all statistically significant findings arose from these, while the three nonsponsored studies reported no benefit. These trends echo those identified in recent meta‐analyses by Myung and Park [20], who showed that collagen's apparent efficacy vanished when limited to independently funded or high‐quality trials. Such findings underscore the persistent risk of sponsorship bias and selective reporting, well‐documented phenomena in pharmaceutical and nutraceutical research [21].

Beyond statistical and funding‐related concerns, broader methodological limitations undermine the field. Many studies are of short duration, typically 8–12 weeks, and therefore may be poorly suited to assess outcomes related to intrinsic or extrinsic skin aging, which may progress over years. If oral collagen supplementation has a clear biological effect, then it may involve mechanistic processes like extracellular matrix remodeling, collagen turnover, or other structural changes in dermal architecture which occur gradually and might be missed over too short a clinical frame. There may be effects a clinician expects to appear by 8 weeks. Equally, there also might be effects that do not manifest by 8 weeks but may be apparent by 12 weeks. In other cases, a longer period might be required to see effects. Thus, a mechanistic justification of when an effect would be expected based on clinical expertise or preexisting data is critical in future work. Without this experimental justification, the question of whether such short duration studies are clinically valuable or not remains, but this is a question beyond the scope of this manuscript. While some studies report modest improvements in hydration or elasticity, the clinical significance of these changes is uncertain. For example, a 5% increase in skin hydration measured via corneometry may not be perceptible to the patient nor translate into a visible reduction in fine lines or textural change. Most trials also fail to report whether outcomes crossed any predefined minimal clinically important difference (MCID), and none included long‐term follow‐up to determine whether transient improvements were sustained beyond supplementation cessation.

Another overlooked but critical factor is regression to the mean. Subjects enrolled into trials may often do so during periods of perceived cosmetic decline, such as seasonal dryness or fatigue‐related skin change, and may naturally improve over time regardless of intervention. This is particularly relevant in small trials with subjective outcomes, where participants and assessors are aware of the trial's goals, and blinding may be suboptimal. Indeed, many of the included studies provided limited detail on placebo formulation. Given that collagen peptides have a distinctive taste, mouthfeel, and nutritional profile, inadequate placebo matching may result in partial or full unblinding. This, combined with participants' expectation of benefit, introduces risk of performance and detection bias, especially for subjective endpoints such as “glow,” “radiance,” or self‐reported improvement in wrinkle severity.

Expectancy effects may also be amplified by the socio‐demographic characteristics of typical study participants. Those with the means and motivation to invest in oral collagen are often simultaneously engaging in other proskin behaviors: daily sunscreen use, prescription, or over‐the‐counter retinoids, antioxidant serums, professional treatments such as microneedling or chemical peels, or energy‐based devices. Few trials controlled for these variables or monitored skincare regimens during the study period. It is therefore plausible that any observed improvement reflects a clustering of health‐seeking behaviors rather than a specific effect of collagen. An additional consideration is the potential contribution of improved nutritional status. Oral collagen is a form of hydrolyzed protein, and supplementation may inadvertently increase overall amino acid availability, particularly in individuals with previously suboptimal dietary intake. Few studies reported baseline protein consumption, and none stratified participants by dietary adequacy. In such cases, modest improvements in skin quality could plausibly reflect enhanced systemic nutrition rather than a targeted dermal mechanism of action. This remains a poorly controlled source of confounding.

These concerns are compounded by analytic flaws in the broader evidence synthesis. Many meta‐analyses conducted multiple unadjusted subgroup analyses (by dose, collagen source, duration, or age), yet none corrected for multiplicity or adjusted confidence intervals accordingly. When sufficient subgroup comparisons are made, the probability of Type I error increases substantially. Without correction, some statistically significant findings are almost certainly false positives. Additionally, none of the existing meta‐analyses incorporated trial sequential analysis (TSA) to assess whether the accumulated sample size was adequate to support the claimed conclusions. Given the modest number of participants across all trials (approximately 1700 collectively), and the frequent borderline *p*‐values, many positive findings are likely to be artefactual. A lack of TSA and multiplicity correction undermines confidence in any “significant” effects emerging from this evidence base [22, 23]. As it stands, many of these analyses and their constituent trials may be considered research waste [24, 25], too poorly conducted to answer their own fundamental research question.

Beyond the literature, the widespread uptake of collagen supplements in clinical and consumer settings reflects factors that extend beyond scientific merit. Collagen's popularity has been driven in part by its intuitive biological narrative, namely, that age‐related collagen loss can be reversed by oral supplementation. This appealing, though mechanistically simplistic, concept has been amplified through direct‐to‐consumer marketing, esthetic conference sponsorship, peer‐to‐peer endorsements, and social media influencer advocacy. Unlike pharmaceuticals, nutraceuticals such as hydrolyzed collagen are not required to demonstrate efficacy in large‐scale trials prior to market release. Early, industry‐sponsored studies reporting modest cosmetic improvements, often in underpowered or methodologically limited designs, were sufficient to support product claims in the absence of regulatory scrutiny. As a result, many clinicians and patients have adopted collagen as a low‐risk intervention, despite a lack of high‐quality, independent evidence. This diffusion of practice underscores the need for rigorous, critical appraisal within dermatology and a cautious approach to integrating commercially promoted supplements into routine care.

Until such evidence emerges, oral collagen supplementation should not be promoted or recommended within dermatological practice. National societies such as the British Association of Dermatologists (BAD), European Academy of Dermatology and Venereology (EADV), and relevant regulatory bodies should consider issuing guidance advising clinicians against endorsing collagen supplements for skin aging in the absence of high‐quality, independent evidence. Collagen may yet have a role in dermatology, but at present, its promotion as a validated antiaging intervention is premature and not scientifically defensible.

## 6 Conclusions

Current evidence does not support the recommendation of oral hydrolyzed collagen as a treatment for skin aging. When studies are grouped by compatible measurement, when analytical and methodological errors are corrected, and when dose, sponsorship, and bias are accounted for, the purported benefits of collagen supplementation do not persist. Any marginal improvements observed appear highly contingent on outcome selection, short‐term fluctuations, and industry affiliation. They are not robust across endpoints, not dose‐responsive, not seen in independent trials, and not demonstrably clinically meaningful. Given the strength of public interest and widespread clinical uptake, further research is warranted. However, future trials must be free from commercial bias, adequately powered, and transparently reported. Studies should prespecify primary outcomes with appropriate MCIDs, control for skincare confounders, match placebos rigorously, and include long‐term follow‐up. Trials should also report full protocols and raw data to allow independent re‐analysis.

## Disclosure

Patient and Public Involvement: Both authors are highly involved in medico‐scientific public outreach and this work stemmed from multiple requests by public and patient partners to ascertain evidence quality for collagen supplementation. It was not appropriate or possible to involve patients or the public in the design, or conduct of the research directly, but results of this analysis will be made available to public for dissemination and discussion.

## Ethics Statement

The authors have nothing to report.

## Consent

The authors have nothing to report.

## Conflicts of Interest

The authors declare no conflicts of interest.

## Supporting information


**Data S1:** jocd71208‐sup‐0001‐Supinfo.rar.

## Data Availability

The basic R code and data sets used for analysis are available with this publication.
